# Supplementary material for: Positive early-life olfactory memory is rooted in the olfactory bulb and triggers large-scale changes beyond the olfactory system
Source: PLoS Biol. 2026 Jul 14;24(7):e3003845. doi: 10.1371/journal.pbio.3003845 (PMC13367741; doi:10.1371/journal.pbio.3003845)
Supplement: S1 Fig — No difference is found between PLAY-O and CTRL-O mice regarding (A) the investigation time in the exploration test, (B) the habituation slope in the habituation test, nor (C) the resulting preference index. Data are represented as data points (one data point per mouse) and mean ± SEM. (DOCX) [file pbio.3003845.s009.docx]

***
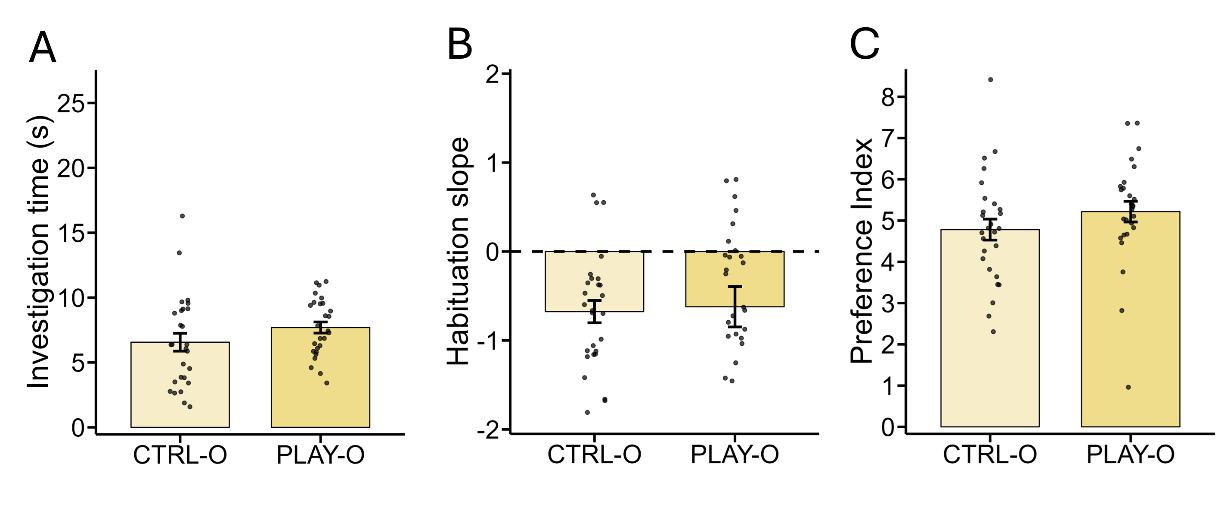
*S1 Fig. Behavioral responses to an unknown odorant in 2-month-old mice (PLAY-O and CTRL-O groups).** No difference is found between PLAY-O and CTRL-O mice regarding (**A**) the investigation time in the exploration test, (**B**) the habituation slope in the habituation test, nor (**C**) the resulting preference index. Data are represented as data points (one data point per mouse) and mean ± SEM (the data underlying this figure can be found in S8 data).
