# Supplementary figures and images for: Positive early-life olfactory memory is rooted in the olfactory bulb and triggers large-scale changes beyond the olfactory system
Source: PLoS Biol. 2026 Jul 14;24(7):e3003845. doi: 10.1371/journal.pbio.3003845 (PMC13367741; doi:10.1371/journal.pbio.3003845)

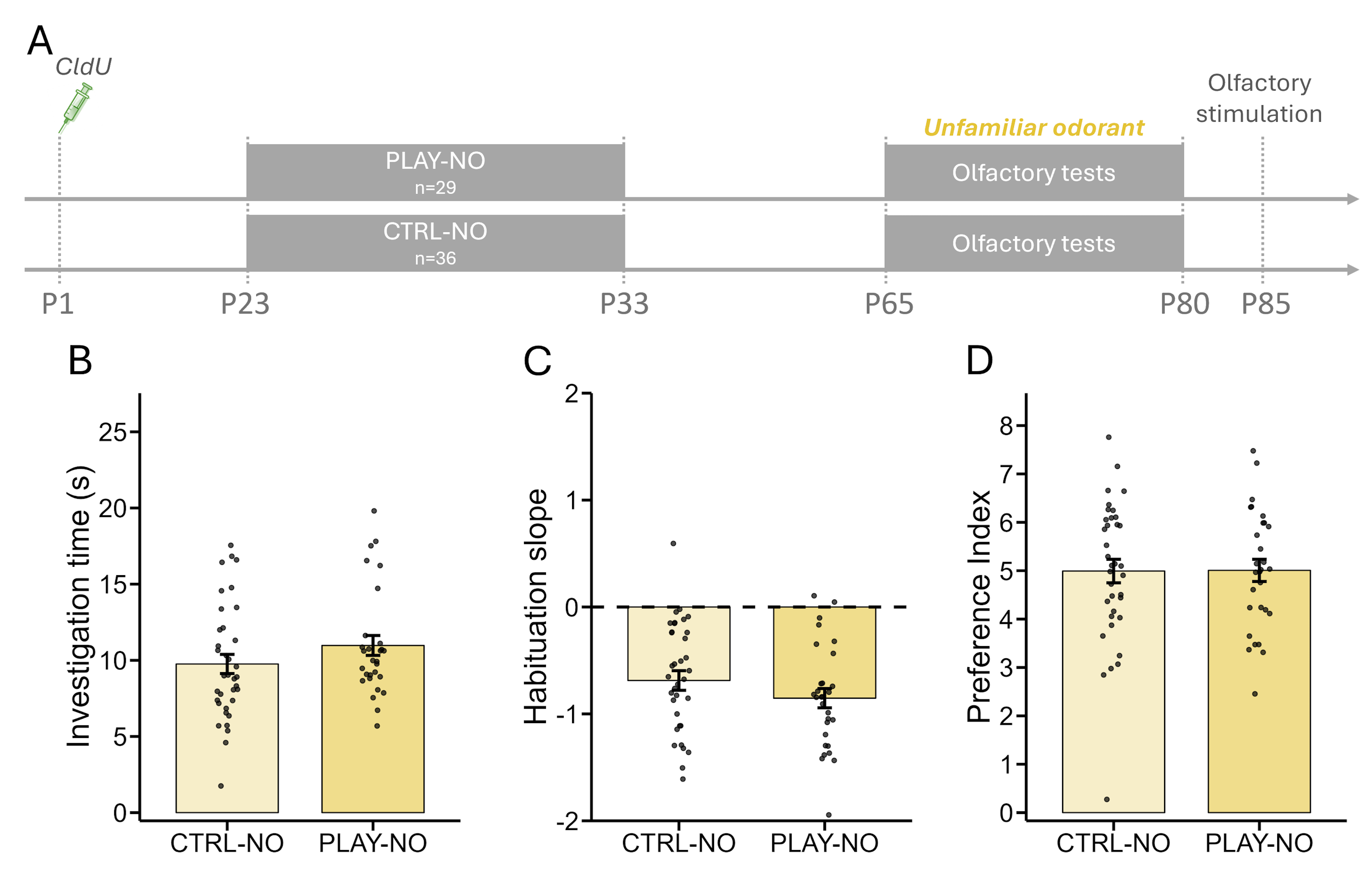

Supplement: S2 Fig — (A) Timeline of the experiment. PLAY-NO (n = 29) and CTRL-NO (n = 36) mice show similar (B) investigation time in the exploration test, (C) habituation slope in the habituation test as well as (D) resulting preference index. Data are represented as data points (one data point per mouse) and mean ± SEM. (TIFF) [file pbio.3003845.s010.tiff]
