# Supplementary material for: Positive early-life olfactory memory is rooted in the olfactory bulb and triggers large-scale changes beyond the olfactory system
Source: PLoS Biol. 2026 Jul 14;24(7):e3003845. doi: 10.1371/journal.pbio.3003845 (PMC13367741; doi:10.1371/journal.pbio.3003845)
Supplement: S3 Fig — (A) The percentage of CldU-positive cells responding to an unknown odorant is similar between PLAY-NO (n = 11) and CTRL-NO (n = 9) mice. Data are represented as data points (one data point per mouse) and mean ± SEM. (DOCX) [file pbio.3003845.s011.docx]

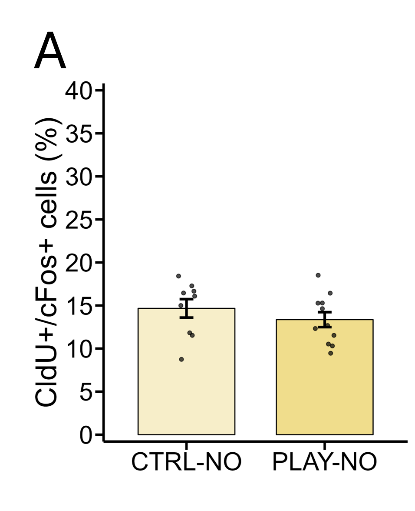
**S3 Fig. Cellular analyses in 2-month-old mice.** (**A**) The percentage of CldU-positive cells responding to an unknown odorant are similar between PLAY-NO (n=11) and CTRL-NO (n=9) mice. Data are represented as data points (one data point per mouse) and mean ± SEM (the data underlying this figure can be found in S8 data).
