# Supplementary material for: Positive early-life olfactory memory is rooted in the olfactory bulb and triggers large-scale changes beyond the olfactory system
Source: PLoS Biol. 2026 Jul 14;24(7):e3003845. doi: 10.1371/journal.pbio.3003845 (PMC13367741; doi:10.1371/journal.pbio.3003845)
Supplement: S4 Fig — The light-induced inhibition of P1-born GCs (PLAY-Halo group, n = 18) does not affect (A) the investigation time, (B) the habituation slope, nor (C) the preference index in response to an unknown odorant, compared to the PLAY-Cont group (n = 12). Data are represented as data points (one data point per mouse) and mean ± SEM. (DOCX) [file pbio.3003845.s012.docx]

**
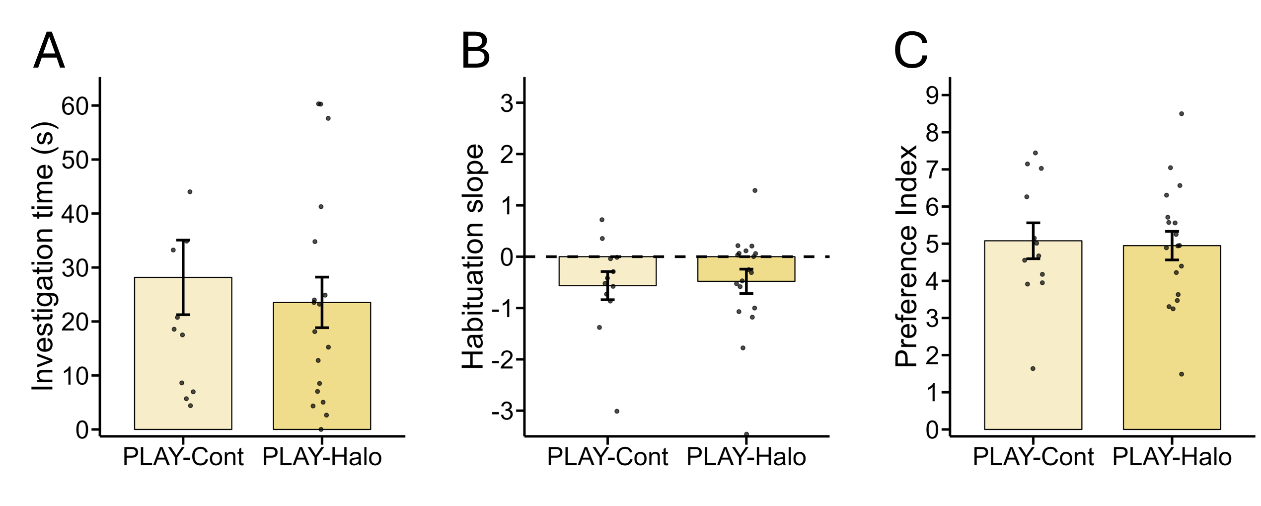
S4 Fig. Behavioral responses to an unknown odorant in the optogenetics experiment.** The light-induced inhibition of P1-born GCs (PLAY-Halo group, n=18) does not affect (**A**) the investigation time, (**B**) the habituation slope, nor (**C**) the preference index in response to an unknown odorant, compared to the PLAY-Cont group (n=12). Data are represented as data points (one data point per mouse) and mean ± SEM (the data underlying this figure can be found in S8 data).
