# Supplementary material for: Positive early-life olfactory memory is rooted in the olfactory bulb and triggers large-scale changes beyond the olfactory system
Source: PLoS Biol. 2026 Jul 14;24(7):e3003845. doi: 10.1371/journal.pbio.3003845 (PMC13367741; doi:10.1371/journal.pbio.3003845)
Supplement: S5 Fig — (A and B) Correlation matrices without p-value thresholding for (A) CTRL-O and (B) PLAY-O groups. Matrix color represents the correlation coefficients between pairs of regions, ranging from −1 to –1. (C and D) Group comparison matrices (PLAY-O – CTRL-O) on (C) nonthresholded matrices to visualize differences in correlation coefficients (scale: −2 to +2; positive values indicate higher correlation coefficients in the PLAY-O group) and on (D) thresholded (p < 0.01) matrices to visualize group-specific correlations (red = specific to PLAY-O, blue = specific to CTRL-O, pink = shared correlations). (E to G) Relative correlation density (i.e., the number of connections normalized by total connections observed in each group) analysis. (E) The relative correlation density is decreased in the olfactory-limbic system and increased in the memory system for PLAY-O compared to CTRL-O group. No significant group differences are observed at the (F) intra-system nor (G) inter-system level. The colored lines represent the subnetworks identified with the Louvain Community approach. Statistical significance depicted as *p < 0.05, **p < 0.01. Abbreviations: AC, accumbens core; ACo, anterior cortical amygdala; AOB, accessory olfactory bulb; AON, anterior olfactory nucleus; AS, accumbens shell; Audi, auditory cortex; BLA, basolateral amygdala; CPu, caudate putamen; dHipp, dorsal hippocampus; GP, globus pallidus; HDB, horizontal limb of the diagonal band of broca; LS, lateral septum; MOB, main olfactory bulb; Mot, motor cortex; mPFC, medial prefrontal cortex; MS, medial septum; OFC, orbitofrontal cortex; Par, parietal cortex; ECx, entorhinal cortex; Pir, piriform cortex; PLCo, posterolateral cortical amygdala; S1, somatosensory cortex 1; S2, somatosensory cortex 2; Tub, olfactory tubercle; TT, tenia tecta; VP, ventral pallidum. (DOCX) [file pbio.3003845.s013.docx]

**
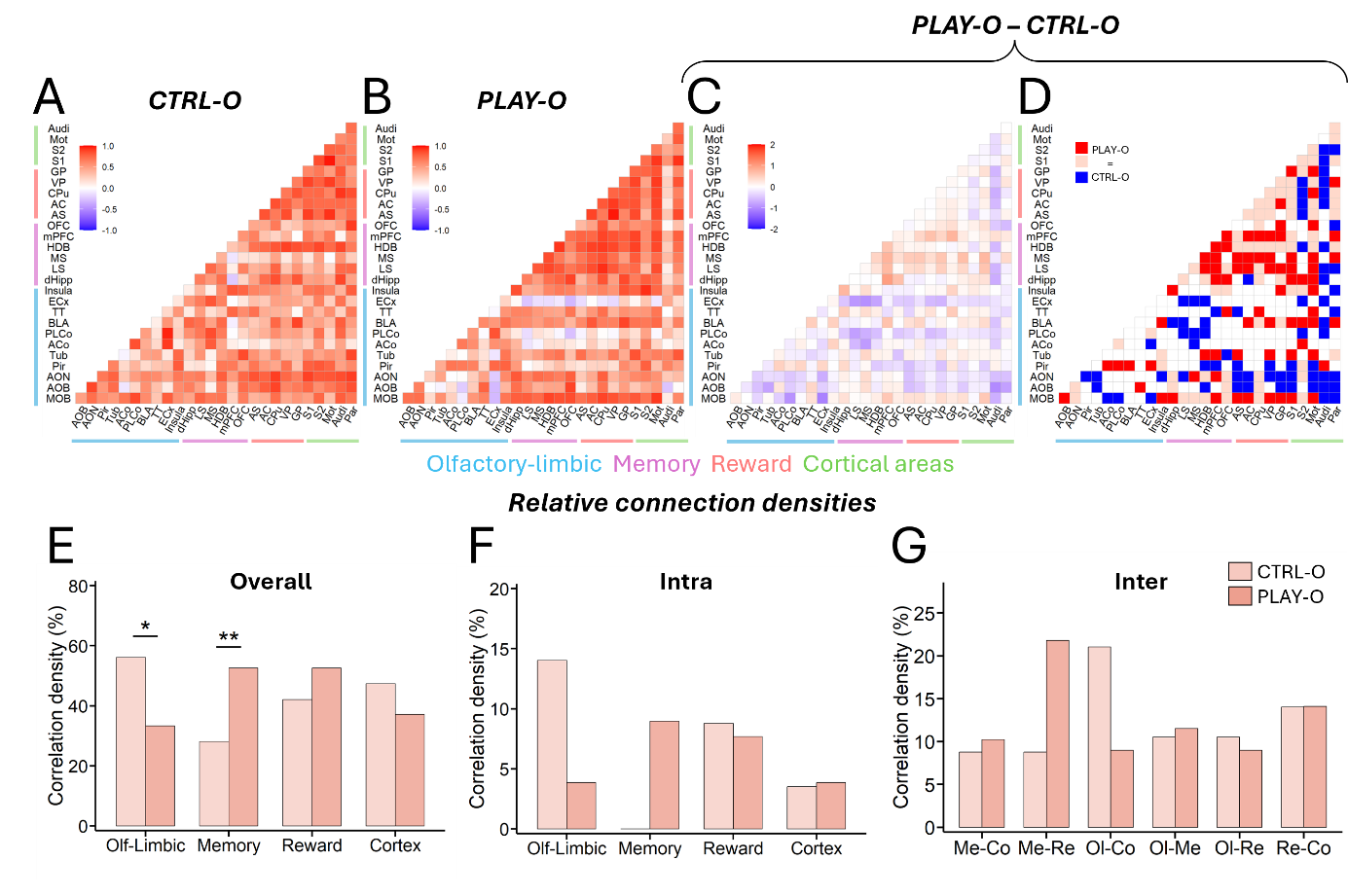
**

**S5 Fig. Functional connectivity analysis in 2-month-old mice (PLAY-O and CTRL-O groups).** (**A** and **B**) Correlation matrices without p-value thresholding for (**A**) CTRL-O and (**B**) PLAY-O groups. Matrix color represents the correlation coefficients between pairs of regions, ranging from -1 to 1. (**C** and **D**) Group comparison matrices (PLAY-O – CTRL-O) on (**C**) non-thresholded matrices to visualize differences in correlation coefficients (scale: -2 to +2; positive values indicate higher correlation coefficients in the PLAY-O group) and on (**D**) thresholded (p < 0.01) matrices to visualize group-specific correlations (red = specific to PLAY-O, blue = specific to CTRL-O, pink = shared correlations). (**E** to **G**) Relative correlation density (i.e., the number of connections normalized by total connections observed in each group) analysis. (**E**) The relative correlation density is decreased in the olfactory-limbic system and increased in the memory system for PLAY-O compared to CTRL-O group. No significant group differences are observed at the (**F**) intra-system nor (**G**) inter-system level. The colored lines represent the subnetworks identified with the Louvain Community approach. Statistical significance depicted as *p < 0.05, **p < 0.01 (the data underlying this figure can be found in S8 data). *AC = Accumbens Core; ACo = Anterior Cortical Amygdala; AOB = Accessory Olfactory Bulb; AON = Anterior Olfactory Nucleus; AS = Accumbens Shell; Audi = Auditory Cortex; BLA = Basolateral Amygdala; CPu = Caudate Putamen; dHipp = dorsal Hippocampus; GP = Globus Pallidus; HDB = Horizontal Limb of the Diagonal Band of Broca; LS = Lateral Septum; MOB = Main Olfactory Bulb; Mot = Motor Cortex; mPFC = medial Prefrontal Cortex; MS = Medial Septum; OFC = Orbitofrontal Cortex; Par = Parietal Cortex; ECx = Entorhinal Cortex; Pir = Piriform Cortex; PLCo = Posterolateral Cortical Amygdala; S1 = Somatosensory Cortex 1; S2 = Somatosensory Cortex 2; Tub = Olfactory Tubercle; TT = Tenia Tecta; VP = Ventral Pallidum.*
