# Supplementary material for: Positive early-life olfactory memory is rooted in the olfactory bulb and triggers large-scale changes beyond the olfactory system
Source: PLoS Biol. 2026 Jul 14;24(7):e3003845. doi: 10.1371/journal.pbio.3003845 (PMC13367741; doi:10.1371/journal.pbio.3003845)
Supplement: S6 Fig — (A) cFos-positive cell density is higher in the dHipp and S1 for PLAY-O (n = 10) compared to PLAY-NO (n = 8) mice. (B to D) Total correlation density analysis (i.e., the number of connections realized over the total possible) analysis. (B) The correlation density is decreased in the olfactory-limbic system for the PLAY-O compared to the PLAY-NO group, reflected by (C) a decrease in the intra-olfactory-limbic system. (D) The PLAY-O group shows increased inter memory-reward correlation density compared to the PLAY-NO group. (E to G) Relative correlation density (i.e., the number of connections normalized by total connections observed in each group) analysis. (E) The relative correlation density is decreased in the olfactory-limbic system and increased in the memory and reward system for the PLAY-O compared to the PLAY-NO group. These differences are reflected by (F) a lower correlation density in the intra-olfactory-limbic system and (G) a higher memory-reward correlation density for the PLAY-O compared to the PLAY-NO group. Statistical significance depicted as *p < 0.05, **p < 0.01. Abbreviations: AC, accumbens core; ACo, anterior cortical amygdala; AOB, accessory olfactory bulb; AON, anterior olfactory nucleus; AS, accumbens shell; Audi, auditory cortex; BLA, basolateral amygdala; CPu, caudate putamen; dHipp, dorsal hippocampus; GP, globus pallidus; HDB, horizontal limb of the diagonal band of broca; LS, lateral septum; MOB, main olfactory bulb; Mot, motor cortex; mPFC, medial prefrontal cortex; MS, medial septum; OFC, orbitofrontal cortex; Par, parietal cortex; ECx, entorhinal cortex; Pir, piriform cortex; PLCo, posterolateral cortical amygdala; S1, somatosensory cortex 1; S2, somatosensory cortex 2; Tub, olfactory tubercle; TT, tenia tecta; VP, ventral pallidum. (DOCX) [file pbio.3003845.s014.docx]

**
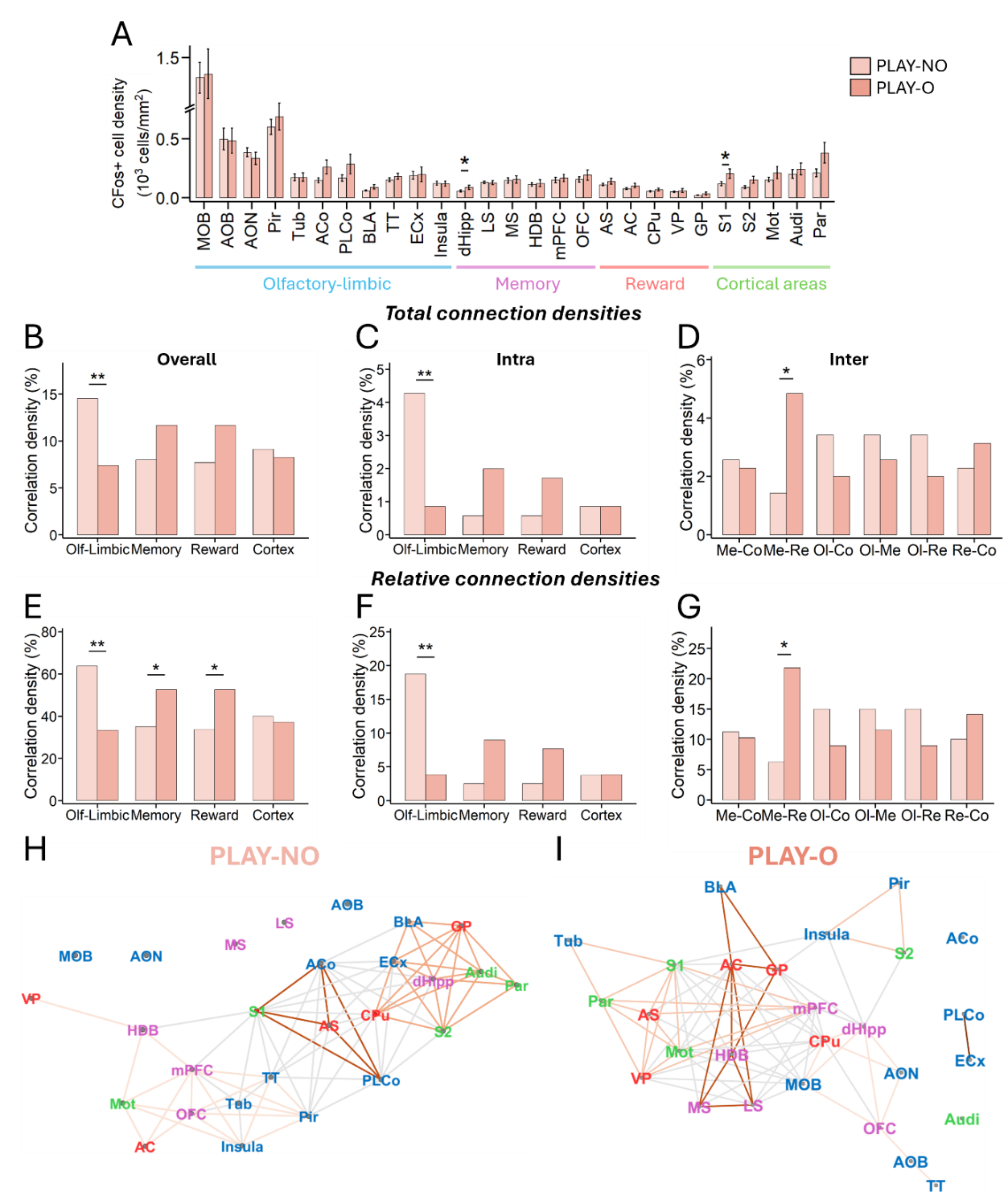
S6 Fig. Functional connectivity analysis in 2-month-old mice (PLAY-O and PLAY-NO groups).** (**A**) cFos-positive cell density is higher in the dHipp and S1 for PLAY-O (n=10) compared to PLAY-NO (n=8) mice. (**B** to **D**) Total correlation density analysis (i.e., the number of connections realized over the total possible) analysis. (**B**) The correlation density is decreased in the olfactory-limbic system for the PLAY-O compared to the PLAY-NO group, reflected by (**C**) a decrease in the intra olfactory-limbic system. (**D**) The PLAY-O group shows increased inter memory-reward correlation density compared to the PLAY-NO group. (**E** to **G**) Relative correlation density (i.e., the number of connections normalized by total connections observed in each group) analysis. (**E**) The relative correlation density is decreased in the olfactory-limbic system and increased in the memory and reward system for the PLAY-O compared to the PLAY-NO group. These differences are reflected by (**F**) a lower correlation density in the intra-olfactory-limbic system and (**G**) a higher memory-reward correlation density for the PLAY-O compared to the PLAY-NO group. Statistical significance depicted as *p < 0.05, **p < 0.01 (the data underlying this figure can be found in S8 data). *AC = Accumbens Core; ACo = Anterior Cortical Amygdala; AOB = Accessory Olfactory Bulb; AON = Anterior Olfactory Nucleus; AS = Accumbens Shell; Audi = Auditory Cortex; BLA = Basolateral Amygdala; CPu = Caudate Putamen; dHipp = dorsal Hippocampus; GP = Globus Pallidus; HDB = Horizontal Limb of the Diagonal Band of Broca; LS = Lateral Septum; MOB = Main Olfactory Bulb; Mot = Motor Cortex; mPFC = medial Prefrontal Cortex; MS = Medial Septum; OFC = Orbitofrontal Cortex; Par = Parietal Cortex; ECx = Entorhinal Cortex; Pir = Piriform Cortex; PLCo = Posterolateral Cortical Amygdala; S1 = Somatosensory Cortex 1; S2 = Somatosensory Cortex 2; Tub = Olfactory Tubercle; TT = Tenia Tecta; VP = Ventral Pallidum.*
