# Supplementary material for: Positive early-life olfactory memory is rooted in the olfactory bulb and triggers large-scale changes beyond the olfactory system
Source: PLoS Biol. 2026 Jul 14;24(7):e3003845. doi: 10.1371/journal.pbio.3003845 (PMC13367741; doi:10.1371/journal.pbio.3003845)
Supplement: S7 Fig — (A to C) Behavioral responses to an unknown odorant. At 6 months of age, PLAY-O (n = 34) and CTRL-O (n = 31) groups show similar (A) habituation slope in the habituation test, (B) investigation time in the exploration test as well as (C) resulting preference index. Data are represented as data points (one data point per mouse) and mean ± SEM. (DOCX) [file pbio.3003845.s015.docx]

**
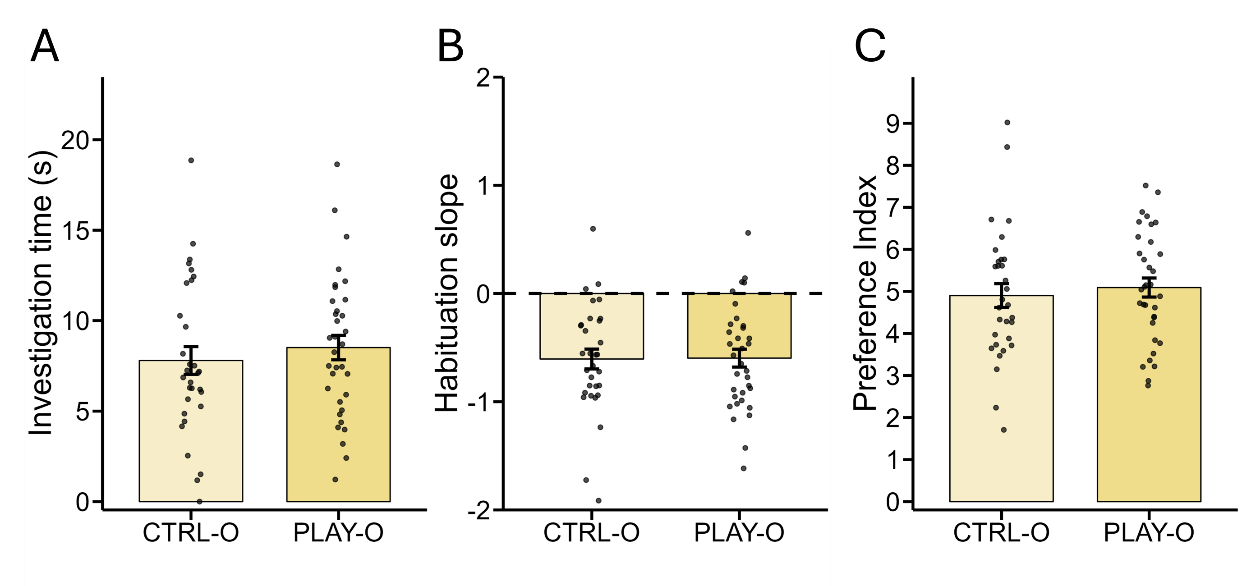
S7 Fig. Behavioral results in 6-month-old mice.** (**A** to **C**) Behavioral responses to an unknown odorant. At 6 months of age, PLAY-O (n=34) and CTRL-O (n=31) groups show similar (**A**) habituation slope in the habituation test, (**B**) investigation time in the exploration test as well as (**C**) resulting preference index. Data are represented as data points (one data point per mouse) and mean ± SEM (the data underlying this figure can be found in S8 data).
