# Supplementary material for: Positive early-life olfactory memory is rooted in the olfactory bulb and triggers large-scale changes beyond the olfactory system
Source: PLoS Biol. 2026 Jul 14;24(7):e3003845. doi: 10.1371/journal.pbio.3003845 (PMC13367741; doi:10.1371/journal.pbio.3003845)
Supplement: S8 Fig — (A to C) Behavioral responses to an unknown odorant. PLAY-rO (n = 29) and CTRL-rO (n = 29) groups show similar (A) investigation time, (B) habituation slope, and (C) preference index in response to an unknown odorant. Data are represented as data points (one data point per mouse) and mean ± SEM. (DOCX) [file pbio.3003845.s016.docx]

**
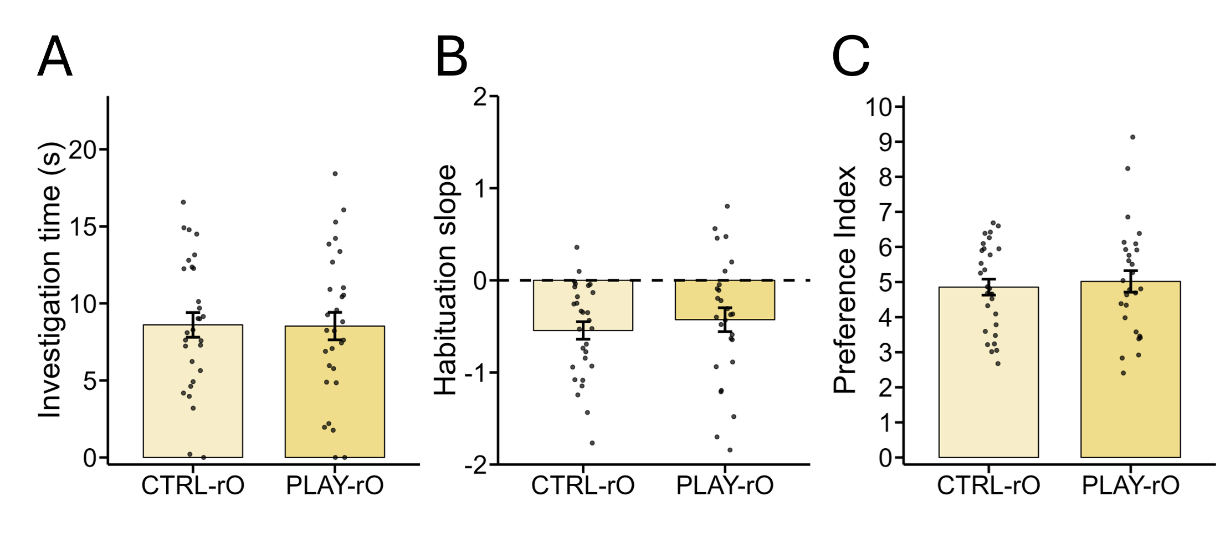
S8 Fig. Behavioral results in 6-month-old mice (with periodic olfactory re-exposures).** (**A** to **C**) Behavioral responses to an unknown odorant. PLAY-rO (n=29) and CTRL-rO (n=29) groups show similar (**A**) investigation time, (**B**) habituation slope and (**C**) preference index in response to an unknown odorant. Data are represented as data points (one data point per mouse) and mean ± SEM (Supp Fig. data).
