# Supplementary material for: Positive early-life olfactory memory is rooted in the olfactory bulb and triggers large-scale changes beyond the olfactory system
Source: PLoS Biol. 2026 Jul 14;24(7):e3003845. doi: 10.1371/journal.pbio.3003845 (PMC13367741; doi:10.1371/journal.pbio.3003845)
Supplement: S9 Fig — (A and B) Correlation matrices without p-value thresholding for (A) CTRL-rO and (B) PLAY-rO groups. Matrix color represents the correlation coefficients between pairs of regions, ranging from −1 to –1. (C and D) Group comparison matrices (PLAY-rO – CTRL-rO) on (C) nonthresholded matrices to visualize differences in correlation coefficients (scale: −2 to +2; positive values indicate higher correlation coefficients in the PLAY-O group) and on (D) thresholded (p < 0.01) matrices to visualize group-specific correlations (red = specific to PLAY-rO, blue = specific to CTRL-rO, pink = shared correlations). (E–G) The relative correlation density (i.e., the number of connections normalized by total connections observed in each group) is (E), increased in the olfactory-limbic system and decreased in the memory and reward systems for the PLAY-rO compared to the CTRL-rO group. These differences are reflected by (F) a higher correlation density in the intra-olfactory-limbic system for the PLAY-rO group as well as (G) a higher olfactory-limbic-cortex and a lower reward-cortex correlation density for the PLAY-rO compared to the CTRL-rO group. Statistical significance depicted as *p < 0.05, **p < 0.01. Abbreviations: AC, accumbens core; ACo, anterior cortical amygdala; AOB, accessory olfactory bulb; AON, anterior olfactory nucleus; AS, accumbens shell; Audi, auditory cortex; BLA, basolateral amygdala; CPu, caudate putamen; dHipp, dorsal hippocampus; GP, globus pallidus; HDB, horizontal limb of the diagonal band of broca; LS, lateral septum; MOB, main olfactory bulb; Mot, motor cortex; mPFC, medial Prefrontal Cortex; MS, medial septum; OFC, orbitofrontal cortex; Par, parietal cortex; ECx, entorhinal cortex; Pir, piriform cortex; PLCo, posterolateral cortical amygdala; S1, somatosensory cortex 1; S2, somatosensory cortex 2; Tub, olfactory tubercle; TT, tenia tecta; VP, ventral pallidum. (DOCX) [file pbio.3003845.s017.docx]

**
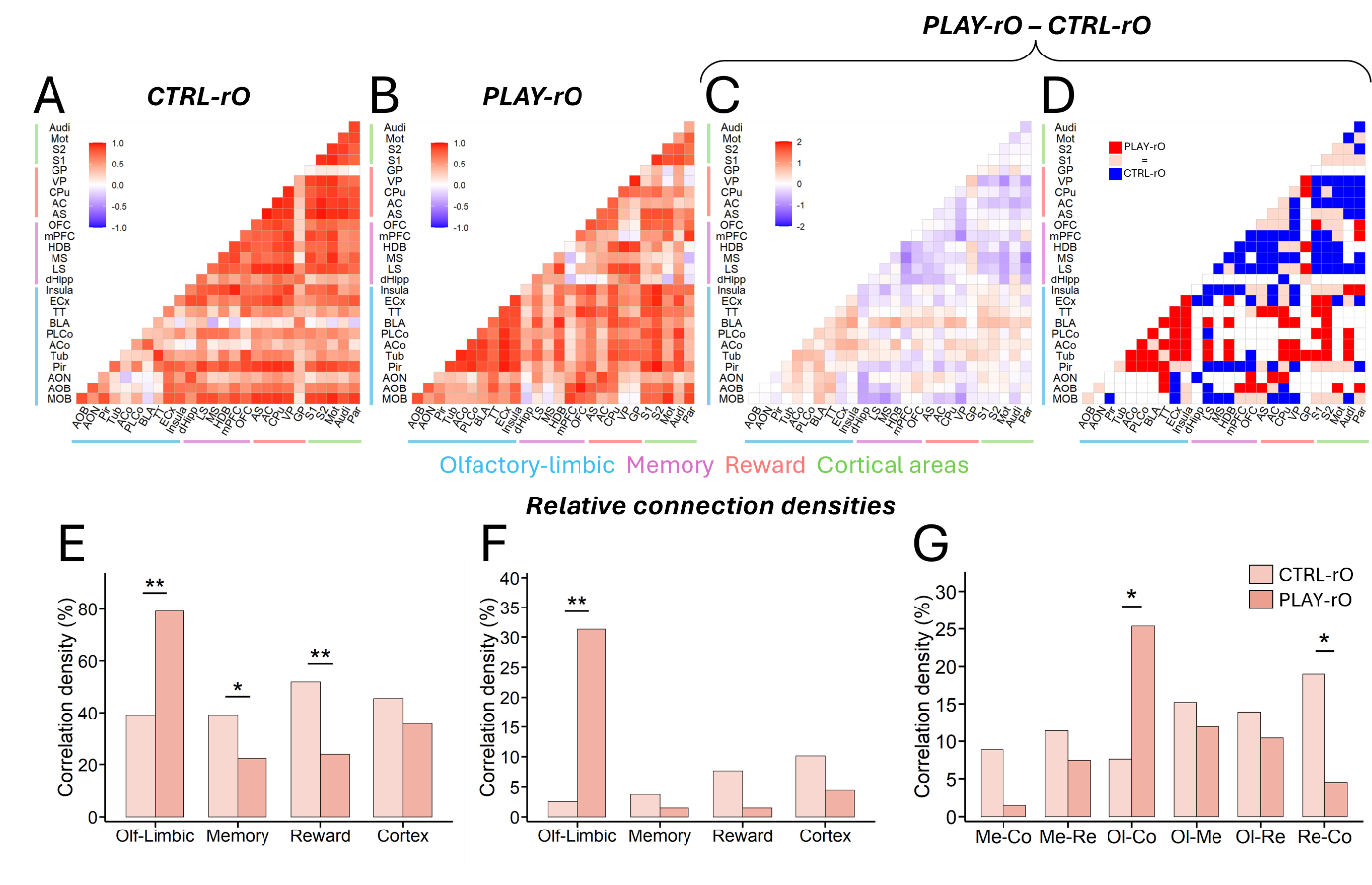
**

**S9 Fig. Functional connectivity analysis in 6-month-old mice (with periodic re-exposures).** (**A** and **B**) Correlation matrices without p-value thresholding for (**A**) CTRL-rO and (**B**) PLAY-rO groups. Matrix color represents the correlation coefficients between pairs of regions, ranging from -1 to 1. (**C** and **D**) Group comparison matrices (PLAY-rO – CTRL-rO) on (**C**) non-thresholded matrices to visualize differences in correlation coefficients (scale: -2 to +2; positive values indicate higher correlation coefficients in the PLAY-O group) and on (**D**) thresholded (p < 0.01) matrices to visualize group-specific correlations (red = specific to PLAY-rO, blue = specific to CTRL-rO, pink = shared correlations). (**E** to **G**) The relative correlation density (i.e., the number of connections normalized by total connections observed in each group) is (**E**), increased in the olfactory-limbic system and decreased in the memory and reward systems for the PLAY-rO compared to the CTRL-rO group. These differences are reflected by (**F**) a higher correlation density in the intra-olfactory-limbic system for the PLAY-rO group as well as (**G**) a higher olfactory-limbic-cortex and a lower reward-cortex correlation density for the PLAY-rO compared to the CTRL-rO group. Statistical significance depicted as *p < 0.05, **p < 0.01 (Supp Fig. data). *AC = Accumbens Core; ACo = Anterior Cortical Amygdala; AOB = Accessory Olfactory Bulb; AON = Anterior Olfactory Nucleus; AS = Accumbens Shell; Audi = Auditory Cortex; BLA = Basolateral Amygdala; CPu = Caudate Putamen; dHipp = dorsal Hippocampus; GP = Globus Pallidus; HDB = Horizontal Limb of the Diagonal Band of Broca; LS = Lateral Septum; MOB = Main Olfactory Bulb; Mot = Motor Cortex; mPFC = medial Prefrontal Cortex; MS = Medial Septum; OFC = Orbitofrontal Cortex; Par = Parietal Cortex; ECx = Entorhinal Cortex; Pir = Piriform Cortex; PLCo = Posterolateral Cortical Amygdala; S1 = Somatosensory Cortex 1; S2 = Somatosensory Cortex 2; Tub = Olfactory Tubercle; TT = Tenia Tecta; VP = Ventral Pallidum.*
