# Supplementary material for: Positive early-life olfactory memory is rooted in the olfactory bulb and triggers large-scale changes beyond the olfactory system
Source: PLoS Biol. 2026 Jul 14;24(7):e3003845. doi: 10.1371/journal.pbio.3003845 (PMC13367741; doi:10.1371/journal.pbio.3003845)
Supplement: S1 Table — (DOCX) [file pbio.3003845.s018.docx]

| **Region** | **MOB** | **AOB** | **AON** | **Pir** | **Tub** | **ACo** | **PLCo** | **BLA** | **TT** | **ECx** | **Insula** | **dHipp** | **LS** | **MS** |
| --- | --- | --- | --- | --- | --- | --- | --- | --- | --- | --- | --- | --- | --- | --- |
| p-value | 0.739 | 0.190 | 0.579 | 0.739 | 0.971 | 0.218 | 0.796 | 0.247 | 0.912 | 0.315 | 0.853 | 0.353 | 0.280 | 0.481 |
| **Region** | **HDB** | **mPFC** | **OFC** | **AS** | **AC** | **CPu** | **VP** | **GP** | **S1** | **S2** | **Mot** | **Audi** | **Par** |  |
| p-value | 0.436 | 0.579 | 0.684 | 0.853 | 0.912 | 0.853 | 0.315 | 0.579 | 0.796 | 0.796 | 0.579 | 0.739 | 0.315 |  |

**S1 Table. Comparison of cFos-positive cell densities between PLAY-O and CTRL-O groups, tested at two months.**
