# Supplementary material for: Positive early-life olfactory memory is rooted in the olfactory bulb and triggers large-scale changes beyond the olfactory system
Source: PLoS Biol. 2026 Jul 14;24(7):e3003845. doi: 10.1371/journal.pbio.3003845 (PMC13367741; doi:10.1371/journal.pbio.3003845)
Supplement: Attachment — Submitted filename: Dejou et al._Responses Reviewers.docx [file pbio.3003845.s026.docx]

We are delighted by the positive evaluation of our manuscript and sincerely grateful to the reviewers for their constructive comments and suggestions. We have carefully addressed all reviewer comments in the revised version of the manuscript. Regarding the additional data requested by Reviewer 1, we fully agree that this is an important point and have addressed it in the manuscript (Supplementary Fig. S1 for behavioral data and Supplementary Fig. S3 for cellular data).

All modifications made in response to the reviewers’ comments are highlighted in red and bold throughout the revised manuscript.

REVIEWS:

**Reviewer #1:** This manuscript describes an exciting model of positive memories evoked by childhood odor experience. Direct and anecdotal evidence exists for such memories and may be a rather unique form of emotional and sensory memory. Here, the authors begin with a simple survey to explore the parameters of human childhood odor memories in adults and use this descriptive data to guide development of a mouse model, including odor characteristics, contextual/reward features and role of repeated exposures. The mouse model thus includes exposure to an attractive odor in a pleasant environment (quantitatively confirmed) during early post-weaning followed by occasional re-exposure and then neurobehavioral testing and manipulations as young adults. Controls include odor exposure in a standard context and no odor exposure in the pleasant or standard contexts. Behavioral results demonstrate a long-lasting attraction to the learned odor. Neural manipulations demonstrate that this long-term memory of the childhood odor requires activity in postnatal day 1 born olfactory bulb granule cells (enhanced fos activation of these cells by the learned odor and optogenetic suppression during testing impairs memory). Brain network activity/functional connectivity assays using fos quantification in sensory, reward, memory, and cortical regions demonstrate selective changes in network connectivity, especially in memory, reward and olfactory/limbic circuits following the learning. These network changes vary depending on the delay between training and testing.

Overall, the design and analyses are excellent, the results novel and impactful, and the manuscript should attract interest in a variety of fields including sensory development, memory and plasticity, and even some translational interests.

In general, this critique offers relatively minor suggestions for changes with one exception. While the design is careful to include a no-odor exposure group to control for effects of enriched environment on these assays, there is no inclusion of the effects of a novel odor at neurobehavioral test. Does an intense, pleasant experience with an odor modify processing of just that odor, or would the effects be generalized? This is particularly relevant in lab animals with minimal novel odor exposures. Would a "pain au chocolat" have been just as effective at evoking a memory as the "Madeline" for a chemosensory restricted Proust? Understanding that adding this control at this point to what is already a substantial piece of work represents a substantial effort, the authors are encouraged to consider it at least in the behavioral experiments or at minimum address this concern in the discussion.

**We thank the referee for this comment. We agree that this is an important issue and was addressed in the manuscript. Specifically, we have clarified the Methods section to better highlight the experiment. All mice were tested with both childhood and unknown odorants, which were all selected to be attractive (Kermen et al 2016; Midroit et al 2021). A set of three attractive odorants was used, with different odorants assigned across animals as either the enriched childhood odorant or the unknown odorant. The results obtained in response to the unknown odorant are presented in Supplementary Fig. S1 for behavioral data and Supplementary Fig. S3 for cellular data. Supp Figure 1 and 3 shows that unknown attractive odorant is not preferred in Play-O versus CTRL-O group and does not recruit P1 neurons.**

Another moderate issue is the frequent description of group differences in the Results that do not seem to reach the standard p <0.05. For example, on page 7 "The childhood odorant is remembered in adulthood" section has several results that appear to be trends but are clearly not < 0.05.

**Indeed, we toned down the sentence regarding the preference for the childhood odorant in the free exploration task showing a p=0.057. The combination score including the habituation task and the preference test shows a significant difference at the level of p=0.0027 between Play-O and CTRL-O8 (p7). In addition, we clarified the presentation of the statistics for Figure S5.**

Minor points

Abstract (and elsewhere) Typically "pleasant" odor should be restricted to human subjects. The more operationally defined "attractive" odor would be better here when talking about mice.

**We have changed “pleasant” to “attractive” when referring to odorants in mice, both in the Abstract and throughout the text.**

Fig. 1 A and D show mean and SEM but no data points as shown in other figures and mentioned in the text

**Data points have been added for the Fig. 1D.
Concerning the Fig. 1A, since responses were measured on a Likert scale (1–9), displaying all 632 datapoints in the figure reduced overall readability and made the distribution difficult to interpret. To provide a clearer visualization of the data while still representing the distribution of individual responses, we replaced the original bar plots with violin plots.**

Fig. 5 would be strengthened by moving the associated no odor control network figure from the Supplement to this figure.

**The no-odor control network (initially presented in Fig. S6H) has been moved to Fig. 5J.**

**Reviewer #2:** The paper from Dejou and colleagues addresses a very important question, namely how early-life experiences shape long-term memory. It does so in an elegant and compelling way, beginning with a human survey and then using those results to inform mechanistic experiments in mice (this cross-species approach is a major strength). The question is highly interesting, the methods are sound, and I strongly support publication in PLOS Biology after minor revisions. It is clear that this is a resubmission, the manuscript is already in excellent shape. My comments below are all relatively minor and mainly concern the inclusion of additional methodological details and clarification of statistical approaches, rather than any major issues.

1.	For the human study, it would be important to specify the language in which the survey was conducted. Was it administered in French? If so, the exact terms used should be reported. This is particularly relevant given the interactions between language, odours, and emotional perception.

**The survey was administered in French. We have reported the original questions, together with their English translations, for clarity.**

2.	In the Methods, odour dilutions are described in terms of vapor pressure, which is fine as it provides a measure of volatility. However, for reproducibility, it would also be helpful to report the liquid dilution, aka percentage of odorant in solvent. More importantly the provider and catalogue number for each odorant should be included.

**We have provided the dilution used to achieve the reported vapor pressure, as well as the catalog number and provider for each odorant.**

3.	For the quantification of adult-born neurons, more detail is needed. How was a cell defined as positive? Were thresholds applied, and if so, how were they determined? How were sections selected, for example across the rostrocaudal, dorseventral, and mediolateral axes of the olfactory bulb? Was counting performed manually or using automated methods?

**For the CldU/cFos and IdU/cFos experiments, cell counting was performed manually, blind to the group condition, and cells were classified as cFos-positive using a low threshold set visually. Six to eight sections spanning the rostrocaudal axis of the left hemisphere were analyzed, with one ×60 magnification acquisition performed in the dorsal region and one in the ventral region of each section.
For CldU and IdU density analyses, cell counting was performed automatically using QuPath software and a machine-learning-based algorithm across the entire granule cell layer of both hemispheres in 3 to 8 sections. We added this information in the method section.**

4.	In the optogenetic experiments, it's important to clarify whether only granule cells were labelled and expressed the reporter, or whether other olfactory bulb cell types born at P1 (e.g. periglomerular cells) were also affected. The specificity of labelling across cell types should be clearly reported.

**We acknowledge that the labeling is not strictly restricted to granule cells. Periglomerular interneurons, some of which are also generated around P1, may indeed be affected by the labeling strategy. We added this information in the method section. In contrast, mitral cells are generated embryonically and are therefore not targeted under our conditions.**

**Importantly, the optical fiber was positioned in the middle of the OB, which preferentially targets deeper layers. As a result, stimulation is expected to predominantly affect granule cells.**

5.	Regarding statistical analysis and figure presentation, it is important to clarify what constitutes the biological replicate, particularly for cell-counting experiments. While it is great to show the spread of data points, figures should also indicate which data points originate from the same animal. Statistical analyses should account for this structure using appropriate nested approaches, such as mixed-effects or multilevel models with animal as a random factor. It is currently unclear whether this has been done, and confirming this would be important, particularly in light of established recommendations in the field (PMID: 24671065).

**For all figures, each data point corresponds to one animal (for cellular analyses, each point represents the mean cell count obtained for a given mouse). Accordingly, classical statistical tests were performed, without the inclusion of models using animal identity as a random factor. For clarity, this information has been specified in the figure legends.**

**Reviewer #3:** This manuscript presents an elegant and ambitious attempt to model childhood olfactory memory in mice and to uncover its neural substrates across the lifespan. The study is conceptually compelling, combining a human survey with a carefully designed behavioral paradigm to capture key features of autobiographical odor memory—namely repeated exposure and positive valence. This cross-species grounding is a particular strength and gives the work a strong translational flavor.

The behavioral data convincingly show that pairing an odor with a positive early-life environment leads to a long-lasting preference in adulthood, supported by multiple complementary assays. The use of ultrasonic vocalizations as a proxy for affective state is appropriate and strengthens the interpretation that the memory carries positive valence. Importantly, the authors go beyond behavior to identify a cellular substrate, showing that P1-born granule cells are preferentially recruited during recall and are causally involved, as demonstrated by optogenetic inhibition. This is a major strength of the study and provides a rare link between early neurogenesis and long-term memory.

At the systems level, the cFos-based functional connectivity analysis is particularly interesting. The identification of a network spanning olfactory, reward, and memory circuits—and its reorganization with age—is intriguing and broadly consistent with the affective and autobiographical nature of odor memories. The shift from hippocampal involvement in young adults to a more olfactory-limbic network later in life is a compelling observation that aligns with ideas of systems consolidation.

That said, several points would benefit from clarification or further discussion. First, the choice of the P23-P33 window as a model of "childhood" in mice is not fully justified. Given that olfaction dominates sensory experience even earlier in development, it would be important to discuss why earlier time points were not considered, and how this choice might influence the conclusions. Second, while the human survey is a nice motivating element, its integration into the experimental design could be more critically evaluated—particularly regarding variability in human experiences versus the relatively constrained mouse paradigm.

**Thank you for allowing us to be more precise. A sentence has been added in the method section: Enrichment was conducted between P23 and P33 corresponding to early childhood as weaning occurs at P21 and sexual maturity around P45.**

**We agree that olfactory experience plays a critical role even earlier in development. To account for your comment, we added this paragraph in the discussion section:**

**We chose the P23–P33, a developmental stage often considered analogous to early childhood in humans, when long-term associative memories such as the “Proust phenomenon” are robustly formed as confirmed by our human survey. Despite the diversity of human experiences leading to the formation of a childhood memory, our model captured the main features of this memory to implement them in mice and revealed their neural basis. Importantly, memory formed during this specific time window does not preclude the possibility that earlier olfactory experiences, including those occurring during the neonatal period, also have strong and lasting effects.**

More conceptually, the authors may want to relate their findings to recent work on "concept cells" and multisensory representations of memory, as this could provide a broader framework for interpreting how odor-specific memories become embedded in distributed networks. Finally, while the connectivity analyses are suggestive, they remain correlational; a more cautious interpretation of "network reorganization" would strengthen the manuscript.

**We thank the reviewer for this stimulating suggestion. We have added the following paragraph in the discussion:
“These findings may be further interpreted in light of the "concept cells" framework *(66)*. These neurons, identified in the human medial temporal lobe, respond to specific concepts regardless of the sensory modality through which they are presented, and are thought to underlie abstract, distributed memory representations. We propose that neonatal bulbar neurogenesis may represent an early developmental mechanism contributing to the formation of such representations. By establishing privileged connections between the olfactory bulb and downstream structures (as identified in our functional connectivity analyses) during a critical developmental window, this initially olfactory network may progressively expand into a multimodal, distributed memory trace, functionally analogous to what concept cells reflect in adult humans. This framework further generates a testable prediction: animals having formed a neonatal olfactory memory should show reactivation of olfactory networks in response to contextual cues alone, even in the absence of the odorant itself, a question we leave open for future research.”**

**We agree that the connectivity analyses are correlational. Accordingly, we have revised the manuscript to adopt a more cautious interpretation of these results by presenting our conclusions as hypothesis.**

Overall, this is a creative and well-executed study that addresses an underexplored but important question. With some clarification and a slightly more nuanced discussion, it will make a valuable contribution to the field.
