# Supplementary material for: Positive early-life olfactory memory is rooted in the olfactory bulb and triggers large-scale changes beyond the olfactory system
Source: PLoS Biol. 2026 Jul 14;24(7):e3003845. doi: 10.1371/journal.pbio.3003845 (PMC13367741; doi:10.1371/journal.pbio.3003845)
Supplement: Peer review history

# Editor report: Decision Letter 0

**DOI:** 10.1371/journal.pbio.3003845.r001

**Authors:** Christian Schnell, PhD

1 Apr 2026

Dear Nathalie,

Thank you for submitting your manuscript entitled "Positive childhood olfactory memory is rooted in the olfactory bulb and triggers large-scale changes beyond the olfactory system" for consideration as a Research Article by PLOS Biology.

Your manuscript has now been evaluated by the PLOS Biology editorial staff and I am writing to let you know that we would like to send your submission out for external peer review.

However, before we can send your manuscript to reviewers, we need you to complete your submission by providing the metadata that is required for full assessment. To this end, please login to Editorial Manager where you will find the paper in the 'Submissions Needing Revisions' folder on your homepage. Please click 'Revise Submission' from the Action Links and complete all additional questions in the submission questionnaire.

Once your full submission is complete, your paper will undergo a series of checks in preparation for peer review. After your manuscript has passed the checks it will be sent out for review. To provide the metadata for your submission, please Login to Editorial Manager (https://www.editorialmanager.com/pbiology) within two working days, i.e. by Apr 07 2026 11:59PM.

If your manuscript has been previously peer-reviewed at another journal, PLOS Biology is willing to work with those reviews in order to avoid re-starting the process. Submission of the previous reviews is entirely optional and our ability to use them effectively will depend on the willingness of the previous journal to confirm the content of the reports and share the reviewer identities. Please note that we reserve the right to invite additional reviewers if we consider that additional/independent reviewers are needed, although we aim to avoid this as far as possible. In our experience, working with previous reviews does save time.

If you would like us to consider previous reviewer reports, please edit your cover letter to let us know and include the name of the journal where the work was previously considered and the manuscript ID it was given. In addition, please upload a response to the reviews as a 'Prior Peer Review' file type, which should include the reports in full and a point-by-point reply detailing how you have or plan to address the reviewers' concerns.

During the process of completing your manuscript submission, you will be invited to opt-in to posting your pre-review manuscript as a bioRxiv preprint. Visit http://journals.plos.org/plosbiology/s/preprints for full details. If you consent to posting your current manuscript as a preprint, please upload a single Preprint PDF.

Feel free to email us at plosbiology@plos.org if you have any queries relating to your submission.

Kind regards,

Christian

Christian Schnell, PhD

Senior Editor

PLOS Biology

cschnell@plos.org

# Peer review: Decision Letter 1

**DOI:** 10.1371/journal.pbio.3003845.r002

**Authors:** Christian Schnell, PhD, Christian Schnell, PhD

30 Apr 2026

Dear Nathalie,

Thank you for your patience while your manuscript "Positive childhood olfactory memory is rooted in the olfactory bulb and triggers large-scale changes beyond the olfactory system" went through peer-review at PLOS Biology. Your manuscript has now been evaluated by the PLOS Biology editors, an Academic Editor with relevant expertise, and by several independent reviewers.

In light of the reviews, which you will find at the end of this email, we are pleased to offer you the opportunity to address the comments from the reviewers in a revision that we anticipate should not take you very long. We will then assess your revised manuscript and your response to the reviewers' comments with our Academic Editor aiming to avoid further rounds of peer-review, although we might need to consult with the reviewers, depending on the nature of the revisions.

You will see in the comments below that Reviewer 1 strongly encourages you to provide an additional dataset. We agree with the reviewer that the question of the specificity of the positive effect is very interesting, but since the result will not fundamentally affect the main point of your manuscript, we do not think that it is absolutely necessary to include this experiment. If you are in a position to provide this dataset, however, I would be happy to extend the revision deadline. Otherwise, you could discuss what is known from the literature on the specificity of sensory enrichment during neonatal development.

In addition to these revisions, you may need to complete some formatting changes, which you will receive in a follow up email. A member of our team will be in touch with a set of requests shortly. If you do not receive a separate email within a few days, please assume that checks have been completed, and no additional changes are required.

We expect to receive your revised manuscript within 1 month. Please email us (plosbiology@plos.org) if you have any questions or concerns, or would like to request an extension.

At this stage, your manuscript remains formally under active consideration at our journal; please notify us by email if you do not intend to submit a revision so that we withdraw the manuscript.

**IMPORTANT - SUBMITTING YOUR REVISION**

Your revisions should address the specific points made by each reviewer. Please submit the following files along with your revised manuscript:

1. A 'Response to Reviewers' file - this should detail your responses to the editorial requests, present a point-by-point response to all of the reviewers' comments, and indicate the changes made to the manuscript.

*NOTE: In your point-by-point response to the reviewers, please provide the full context of each review. Do not selectively quote paragraphs or sentences to reply to. The entire set of reviewer comments should be present in full and each specific point should be responded to individually.

You should also cite any additional relevant literature that has been published since the original submission and mention any additional citations in your response.

2. In addition to a clean copy of the manuscript, please also upload a 'track-changes' version of your manuscript that specifies the edits made. This should be uploaded as a "Revised Article with Changes Highlighted " file type.

*Resubmission Checklist*

When you are ready to resubmit your revised manuscript, please refer to this resubmission checklist: https://plos.io/Biology_Checklist

To submit a revised version of your manuscript, please go to https://www.editorialmanager.com/pbiology/ and log in as an Author. Click the link labelled 'Submissions Needing Revision' where you will find your submission record.

Please make sure to read the following important policies and guidelines while preparing your revision:

*Published Peer Review*

Please note while forming your response, if your article is accepted, you may have the opportunity to make the peer review history publicly available. The record will include editor decision letters (with reviews) and your responses to reviewer comments. If eligible, we will contact you to opt in or out. Please see here for more details:

https://blogs.plos.org/plos/2019/05/plos-journals-now-open-for-published-peer-review/

*PLOS Data Policy*

Please note that as a condition of publication PLOS' data policy (http://journals.plos.org/plosbiology/s/data-availability) requires that you make available all data used to draw the conclusions arrived at in your manuscript. If you have not already done so, you must include any data used in your manuscript either in appropriate repositories, within the body of the manuscript, or as supporting information (N.B. this includes any numerical values that were used to generate graphs, histograms etc.). For an example see here: http://www.plosbiology.org/article/info%3Adoi%2F10.1371%2Fjournal.pbio.1001908#s5

*Blot and Gel Data Policy*

We require the original, uncropped and minimally adjusted images supporting all blot and gel results reported in an article's figures or Supporting Information files. We will require these files before a manuscript can be accepted so please prepare them now, if you have not already uploaded them. Please carefully read our guidelines for how to prepare and upload this data: https://journals.plos.org/plosbiology/s/figures#loc-blot-and-gel-reporting-requirements

*Protocols deposition*

To enhance the reproducibility of your results, we recommend that if applicable you deposit your laboratory protocols in protocols.io, where a protocol can be assigned its own identifier (DOI) such that it can be cited independently in the future. Additionally, PLOS ONE offers an option for publishing peer-reviewed Lab Protocol articles, which describe protocols hosted on protocols.io. Read more information on sharing protocols at https://plos.org/protocols?utm_medium=editorial-email&utm_source=authorletters&utm_campaign=protocols

Thank you again for your submission to our journal. We hope that our editorial process has been constructive thus far, and we welcome your feedback at any time. Please don't hesitate to contact us if you have any questions or comments.

Sincerely,

Christian

Christian Schnell, PhD

Senior Editor

PLOS Biology

cschnell@plos.org

----------------------------------------------------------------

REVIEWS:

Reviewer #1: This manuscript describes an exciting model of positive memories evoked by childhood odor experience. Direct and anecdotal evidence exists for such memories and may be a rather unique form of emotional and sensory memory. Here, the authors begin with a simple survey to explore the parameters of human childhood odor memories in adults and use this descriptive data to guide development of a mouse model, including odor characteristics, contextual/reward features and role of repeated exposures. The mouse model thus includes exposure to an attractive odor in a pleasant environment (quantitatively confirmed) during early post-weaning followed by occasional re-exposure and then neurobehavioral testing and manipulations as young adults. Controls include odor exposure in a standard context and no odor exposure in the pleasant or standard contexts. Behavioral results demonstrate a long-lasting attraction to the learned odor. Neural manipulations demonstrate that this long-term memory of the childhood odor requires activity in postnatal day 1 born olfactory bulb granule cells (enhanced fos activation of these cells by the learned odor and optogenetic suppression during testing impairs memory). Brain network activity/functional connectivity assays using fos quantification in sensory, reward, memory, and cortical regions demonstrate selective changes in network connectivity, especially in memory, reward and olfactory/limbic circuits following the learning. These network changes vary depending on the delay between training and testing.

Overall, the design and analyses are excellent, the results novel and impactful, and the manuscript should attract interest in a variety of fields including sensory development, memory and plasticity, and even some translational interests.

In general, this critique offers relatively minor suggestions for changes with one exception. While the design is careful to include a no-odor exposure group to control for effects of enriched environment on these assays, there is no inclusion of the effects of a novel odor at neurobehavioral test. Does an intense, pleasant experience with an odor modify processing of just that odor, or would the effects be generalized? This is particularly relevant in lab animals with minimal novel odor exposures. Would a "pain au chocolat" have been just as effective at evoking a memory as the "Madeline" for a chemosensory restricted Proust? Understanding that adding this control at this point to what is already a substantial piece of work represents a substantial effort, the authors are encouraged to consider it at least in the behavioral experiments or at minimum address this concern in the discussion.

Another moderate issue is the frequent description of group differences in the Results that do not seem to reach the standard p <0.05. For example, on page 7 "The childhood odorant is remembered in adulthood" section has several results that appear to be trends but are clearly not < 0.05.

Minor points

Abstract (and elsewhere) Typically "pleasant" odor should be restricted to human subjects. The more operationally defined "attractive" odor would be better here when talking about mice.

Fig. 1 A and D show mean and SEM but no data points as shown in other figures and mentioned in the text

Fig. 5 would be strengthened by moving the associated no odor control network figure from the Supplement to this figure.

Reviewer #2: The paper from Dejou and colleagues addresses a very important question, namely how early-life experiences shape long-term memory. It does so in an elegant and compelling way, beginning with a human survey and then using those results to inform mechanistic experiments in mice (this cross-species approach is a major strength). The question is highly interesting, the methods are sound, and I strongly support publication in PLOS Biology after minor revisions. It is clear that this is a resubmission, the manuscript is already in excellent shape. My comments below are all relatively minor and mainly concern the inclusion of additional methodological details and clarification of statistical approaches, rather than any major issues.

1.For the human study, it would be important to specify the language in which the survey was conducted. Was it administered in French? If so, the exact terms used should be reported. This is particularly relevant given the interactions between language, odours, and emotional perception.

2.In the Methods, odour dilutions are described in terms of vapor pressure, which is fine as it provides a measure of volatility. However, for reproducibility, it would also be helpful to report the liquid dilution, aka percentage of odorant in solvent. More importantly the provider and catalogue number for each odorant should be included.

3.For the quantification of adult-born neurons, more detail is needed. How was a cell defined as positive? Were thresholds applied, and if so, how were they determined? How were sections selected, for example across the rostrocaudal, dorseventral, and mediolateral axes of the olfactory bulb? Was counting performed manually or using automated methods?

4.In the optogenetic experiments, it's important to clarify whether only granule cells were labelled and expressed the reporter, or whether other olfactory bulb cell types born at P1 (e.g. periglomerular cells) were also affected. The specificity of labelling across cell types should be clearly reported.

5.Regarding statistical analysis and figure presentation, it is important to clarify what constitutes the biological replicate, particularly for cell-counting experiments. While it is great to show the spread of data points, figures should also indicate which data points originate from the same animal. Statistical analyses should account for this structure using appropriate nested approaches, such as mixed-effects or multilevel models with animal as a random factor. It is currently unclear whether this has been done, and confirming this would be important, particularly in light of established recommendations in the field (PMID: 24671065).

Reviewer #3: This manuscript presents an elegant and ambitious attempt to model childhood olfactory memory in mice and to uncover its neural substrates across the lifespan. The study is conceptually compelling, combining a human survey with a carefully designed behavioral paradigm to capture key features of autobiographical odor memory—namely repeated exposure and positive valence. This cross-species grounding is a particular strength and gives the work a strong translational flavor.

The behavioral data convincingly show that pairing an odor with a positive early-life environment leads to a long-lasting preference in adulthood, supported by multiple complementary assays. The use of ultrasonic vocalizations as a proxy for affective state is appropriate and strengthens the interpretation that the memory carries positive valence. Importantly, the authors go beyond behavior to identify a cellular substrate, showing that P1-born granule cells are preferentially recruited during recall and are causally involved, as demonstrated by optogenetic inhibition. This is a major strength of the study and provides a rare link between early neurogenesis and long-term memory.

At the systems level, the cFos-based functional connectivity analysis is particularly interesting. The identification of a network spanning olfactory, reward, and memory circuits—and its reorganization with age—is intriguing and broadly consistent with the affective and autobiographical nature of odor memories. The shift from hippocampal involvement in young adults to a more olfactory-limbic network later in life is a compelling observation that aligns with ideas of systems consolidation.

That said, several points would benefit from clarification or further discussion. First, the choice of the P23-P33 window as a model of "childhood" in mice is not fully justified. Given that olfaction dominates sensory experience even earlier in development, it would be important to discuss why earlier time points were not considered, and how this choice might influence the conclusions. Second, while the human survey is a nice motivating element, its integration into the experimental design could be more critically evaluated—particularly regarding variability in human experiences versus the relatively constrained mouse paradigm.

More conceptually, the authors may want to relate their findings to recent work on "concept cells" and multisensory representations of memory, as this could provide a broader framework for interpreting how odor-specific memories become embedded in distributed networks. Finally, while the connectivity analyses are suggestive, they remain correlational; a more cautious interpretation of "network reorganization" would strengthen the manuscript.

Overall, this is a creative and well-executed study that addresses an underexplored but important question. With some clarification and a slightly more nuanced discussion, it will make a valuable contribution to the field.

# Author comment: Author response to Decision Letter 2

**DOI:** 10.1371/journal.pbio.3003845.r003

15 May 2026

Attachment Submitted filename: Dejou et al._Responses Reviewers.docx

# Editor report: Decision Letter 2

**DOI:** 10.1371/journal.pbio.3003845.r004

**Authors:** Christian Schnell, PhD, Christian Schnell, PhD, Christian Schnell, PhD

20 May 2026

Dear Nathalie,

Thank you for your patience while we considered your revised manuscript "Positive childhood olfactory memory is rooted in the olfactory bulb and triggers large-scale changes beyond the olfactory system" for publication as a Research Article at PLOS Biology. This revised version of your manuscript has been evaluated by the PLOS Biology editors and the Academic Editor.

Based on our Academic Editor's assessment of your revision, we are likely to accept this manuscript for publication, provided you satisfactorily address the following data and other policy-related requests:

* We would like to suggest a different title to improve its accessibility for our broad audience:

Positive early-life olfactory memory is rooted in the olfactory bulb and triggers large-scale changes beyond the olfactory system

* Please add the links to the funding agencies in the Financial Disclosure statement in the manuscript details.

* Please include information in the Methods section whether the study has been conducted according to the principles expressed in the Declaration of Helsinki.

* DATA POLICY:

You may be aware of the PLOS Data Policy, which requires that all data be made available without restriction: http://journals.plos.org/plosbiology/s/data-availability. For more information, please also see this editorial: http://dx.doi.org/10.1371/journal.pbio.1001797

Note that we do not require all raw data. Rather, we ask that all individual quantitative observations that underlie the data summarized in the figures and results of your paper be made available in one of the following forms:

1) Supplementary files (e.g., excel). Please ensure that all data files are uploaded as 'Supporting Information' and are invariably referred to (in the manuscript, figure legends, and the Description field when uploading your files) using the following format verbatim: S1 Data, S2 Data, etc. Multiple panels of a single or even several figures can be included as multiple sheets in one excel file that is saved using exactly the following convention: S1_Data.xlsx (using an underscore).

2) Deposition in a publicly available repository. Please also provide the accession code or a reviewer link so that we may view your data before publication.

Regardless of the method selected, please ensure that you provide the individual numerical values that underlie the summary data displayed in the following figure panels as they are essential for readers to assess your analysis and to reproduce it: 1ABD, 2BCDEFG, 3ABC, 4BCDFG, 5BEFG, 6BCDEFHIJK, 7ADEF, S1ABC, S2BCD, S3, S4ABC, S5EFG, S6A-G, S7ABC, S8ABC and S9EFG (I think you have provided this already, but please double-check).

NOTE: the numerical data provided should include all replicates AND the way in which the plotted mean and errors were derived.

Please also ensure that figure legends in your manuscript include information on where the underlying data can be found, and ensure your supplemental data file/s has a legend.

Please ensure that your Data Statement in the submission system accurately describes where your data can be found.

* CODE POLICY

Per journal policy, if you have generated any custom code during the course of this investigation, please make it available without restrictions. Please ensure that the code is sufficiently well documented and reusable, and that your Data Statement in the Editorial Manager submission system accurately describes where your code can be found. More information on our Code Policy, what and how to share can be found here: https://journals.plos.org/plosbiology/s/code-availability

Please note that we cannot accept sole deposition of code in GitHub, as this could be changed after publication. However, you can archive this version of your publicly available GitHub code to Zenodo. Once you do this, it will generate a DOI number, which you will need to provide in the Data Accessibility Statement (you are welcome to also provide the GitHub access information). See the process for doing this here: https://docs.github.com/en/repositories/archiving-a-github-repository/referencing-and-citing-content

As you address these items, please take this last chance to review your reference list to ensure that it is complete and correct. If you have cited papers that have been retracted, please include the rationale for doing so in the manuscript text, or remove these references and replace them with relevant current references. Any changes to the reference list should be mentioned in the cover letter that accompanies your revised manuscript.

In addition to these revisions, you may need to complete some formatting changes, which you will receive in a follow up email. A member of our team will be in touch with a set of requests shortly. If you do not receive a separate email within a few days, please assume that checks have been completed, and no additional changes are required.

We expect to receive your revised manuscript within two weeks.

To submit your revision, please go to https://www.editorialmanager.com/pbiology/ and log in as an Author. Click the link labelled 'Submissions Needing Revision' to find your submission record. Your revised submission must include the following:

- a cover letter that should detail your responses to any editorial requests, if applicable, and whether changes have been made to the reference list

- a Response to Reviewers file that provides a detailed response to the reviewers' comments (if applicable, if not applicable please do not delete your existing 'Response to Reviewers' file.)

- a track-changes file indicating any changes that you have made to the manuscript.

NOTE: If Supporting Information files are included with your article, note that these are not copyedited and will be published as they are submitted. Please ensure that these files are legible and of high quality (at least 300 dpi) in an easily accessible file format. For this reason, please be aware that any references listed in an SI file will not be indexed. For more information, see our Supporting Information guidelines:

https://journals.plos.org/plosbiology/s/supporting-information

*Published Peer Review History*

Please note that you may have the opportunity to make the peer review history publicly available. The record will include editor decision letters (with reviews) and your responses to reviewer comments. If eligible, we will contact you to opt in or out. Please see here for more details:

https://plos.org/published-peer-review-history/

*Press*

Should you, your institution's press office or the journal office choose to press release your paper, please ensure you have opted out of Early Article Posting on the submission form. We ask that you notify us as soon as possible if you or your institution is planning to press release the article.

*Protocols deposition*

To enhance the reproducibility of your results, we recommend that if applicable you deposit your laboratory protocols in protocols.io, where a protocol can be assigned its own identifier (DOI) such that it can be cited independently in the future. Additionally, PLOS ONE offers an option for publishing peer-reviewed Lab Protocol articles, which describe protocols hosted on protocols.io. Read more information on sharing protocols at https://plos.org/protocols?utm_medium=editorial-email&utm_source=authorletters&utm_campaign=protocols

Please do not hesitate to contact me should you have any questions.

Sincerely,

Christian

Christian Schnell, PhD

Senior Editor

cschnell@plos.org

PLOS Biology

# Author comment: Author response to Decision Letter 3

**DOI:** 10.1371/journal.pbio.3003845.r005

22 May 2026

Attachment Submitted filename: Dejou_et_al._Responses_Reviewers_auresp_3.docx

# Editor report: Decision Letter 3

**DOI:** 10.1371/journal.pbio.3003845.r006

**Authors:** Christian Schnell, PhD, Christian Schnell, PhD, Christian Schnell, PhD, Christian Schnell, PhD

26 May 2026

Dear Nathalie,

Thank you for the submission of your revised Research Article "Positive early-life olfactory memory is rooted in the olfactory bulb and triggers large-scale changes beyond the olfactory system" for publication in PLOS Biology. On behalf of my colleagues and the Academic Editor, Izumi Fukunaga, I am pleased to say that we can in principle accept your manuscript for publication, provided you address any remaining formatting and reporting issues. These will be detailed in an email you should receive within 2-3 business days from our colleagues in the journal operations team; no action is required from you until then. Please note that we will not be able to formally accept your manuscript and schedule it for publication until you have completed any requested changes.

While you attend to those requests to come, please also ensure to mention in the figure legends where the source data can be found. For example: "The source data underlying this Figure can be found in S1 Data."

Please take a minute to log into Editorial Manager at http://www.editorialmanager.com/pbiology/, click the "Update My Information" link at the top of the page, and update your user information to ensure an efficient production process.

PRESS

We frequently collaborate with press offices. If your institution or institutions have a press office, please notify them about your upcoming paper at this point, to enable them to help maximise its impact. If the press office is planning to promote your findings, we would be grateful if they could coordinate with biologypress@plos.org. If you have previously opted in to the early version process, we ask that you notify us immediately of any press plans so that we may opt out on your behalf.

We also ask that you take this opportunity to read our Embargo Policy regarding the discussion, promotion and media coverage of work that is yet to be published by PLOS. As your manuscript is not yet published, it is bound by the conditions of our Embargo Policy. Please be aware that this policy is in place both to ensure that any press coverage of your article is fully substantiated and to provide a direct link between such coverage and the published work. For full details of our Embargo Policy, please visit http://www.plos.org/about/media-inquiries/embargo-policy/.

Thank you again for choosing PLOS Biology for publication and supporting Open Access publishing. We look forward to publishing your study.

Sincerely,

Christian

Christian Schnell, PhD

Senior Editor

PLOS Biology

cschnell@plos.org
